# Food patterns and dietary quality associated with organic food consumption during pregnancy; data from a large cohort of pregnant women in Norway

**DOI:** 10.1186/1471-2458-12-612

**Published:** 2012-08-06

**Authors:** Hanne Torjusen, Geir Lieblein, Tormod Næs, Margaretha Haugen, Helle Margrete Meltzer, Anne Lise Brantsæter

**Affiliations:** 1 Division of Environmental Medicine, Norwegian Institute of Public Health, Oslo, Norway; 2 National Institute for Consumer Research (SIFO), Oslo, Norway; 3 Department of Plant and Environmental Sciences, Norwegian University of Life Sciences, Ås, Norway; 4 Nofima, Osloveien 1, 1430, Ås, Norway

## Abstract

**Background:**

Little is known about the consumption of organic food during pregnancy. The aim of this study was to describe dietary characteristics associated with frequent consumption of organic food among pregnant women participating in the Norwegian Mother and Child Cohort Study (MoBa).

**Methods:**

The present study includes 63 808 women who during the years 2002–2007 answered two questionnaires, a general health questionnaire at gestational weeks 15 and a food frequency questionnaire at weeks 17-22. The exploration of food patterns by Principal component analyses (PCA) was followed by ANOVA analyses investigating how these food patterns as well as intake of selected food groups were associated with consumption of organic food.

**Results:**

The first principal component (PC1) identified by PCA, accounting for 12% of the variation, was interpreted as a ‘health and sustainability component’, with high positive loadings for vegetables, fruit and berries, cooking oil, whole grain bread and cereal products and negative loadings for meat, including processed meat, white bread, and cakes and sweets. Frequent consumption of organic food, which was reported among 9.1% of participants (n = 5786), was associated with increased scores on the ‘health and sustainability component’ (p < 0.001). The increase in score represented approximately 1/10 of the total variation and was independent of sociodemographic and lifestyle characteristics. Participants with frequent consumption of organic food had a diet with higher density of fiber and most nutrients such as folate, beta-carotene and vitamin C, and lower density of sodium compared to participants with no or low organic consumption.

**Conclusion:**

The present study showed that pregnant Norwegian women reporting frequent consumption of organically produced food had dietary pattern and quality more in line with public advice for healthy and sustainable diets. A methodological implication is that the overall diet needs to be included in future studies of potential health outcomes related to consumption of organic food during pregnancy.

## Background

The essential importance of dietary quality during pregnancy for the health of mother and child is continuously becoming clearer, and there is growing evidence that maternal diet may influence longer-term health of their children even within relatively well-nourished populations [1-3].

Dietary composition, the kinds of foods that are eaten and their relative amounts, is together with the nutritional quality of food decisive for the impact of the diet on health. Organic production methods are generally claimed to enhance food quality, and research indicating superior quality of organically produced food include findings of higher levels of vitamins, antioxidants [4,5], poly-unsaturated fatty acids [6], and lower levels of heavy metals [7], mycotoxins [8] and pesticide residues [9,10] in a range of crops and/or milk. This matter is far from settled, however, and Dangour et al. [11] suggest, based on a review, that there is not sufficient evidence to draw conclusions about major differences in nutritional quality between organically and conventionally produced food.

In studies from Great Britain, Denmark and Norway, Denver et al. [12], Holt [13] and Torjusen et al. [14] have found associations between the choice of organically produced food and dietary patterns high in fruit and vegetables and low in meat compared to intake levels among consumers who did not buy organic food. It is known from a number of studies that health is a main motivation for choosing organic foods, together with ethical and environmental considerations [15,16]. Since there is a considerable overlap between dietary recommendations aiming for good health and those aiming for environmental sustainability [17], it is possible to theorize that the choice of organic food for both of these reasons may be accompanied by dietary patterns more in line with health recommendations.

Variation in dietary patterns has been described among pregnant women in several populations, including Norway [18-20]. However, there is little knowledge about the diets of women who choose organically grown food during pregnancy. Given the possibility that the choice of organic food may be related to dietary characteristics of relevance to health, and the particular importance of health during pregnancy, it is important to gain more knowledge about this issue. The Norwegian Mother and Child Cohort Study (MoBa), which is a nation-wide pregnancy cohort [21], includes questions about the frequency of organic food consumption, and thus provides an opportunity to explore possible associations between consumption of organically produced food and dietary composition. We have previously reported socio-economic and lifestyle characteristics associated with frequent consumption of organic food among the MoBa participants. The socioeconomic characteristics of pregnant women with frequent organic consumption did not unambiguously follow those typically associated with better health, such as higher education and income [22]. We also plan to investigate potential diet-health relationships related to consumption of organic food.

The aim of the present study was to examine associations between frequent consumption of organically produced food and the overall dietary patterns, food intake and nutrient density of pregnant women in MoBa. In particular, we wanted to investigate whether there were significant differences in dietary patterns between women who frequently ate organic food during pregnancy and those who seldom or never ate organic food. Furthermore, we wanted to investigate whether there were significant differences between consumption of organic food of different food categories (milk/dairy, bread/cereal, eggs, vegetables, fruit and meat) and diet in general.

## Methods

### Subjects and methods

The data set is part of the MoBa study, which is initiated by and maintained at the Norwegian Institute of Public Health. The cohort now includes 108 000 children, 90 700 mothers and 71 500 fathers [21]. Pregnant women were recruited to the study by postal invitation after they had signed up for the routine ultrasound examination in their local hospital [21]. A total of 38.5% of invited women participated in the study. The women were asked to provide biological samples and to answer questionnaires covering a wide range of information. The cohort database is linked to the Medical Birth Registry of Norway [23]. The MoBa study has been approved by the Regional Committee for Ethics in Medical Research and the Data Inspectorate in Norway, and informed written consent was obtained from all participants.

### Subjects

This study uses version 4 of the data files made available for research in January 2009. We only included the first participation for women who were in MoBa with more than one pregnancy. The source population for the present study comprised 65,563 women who had responded to the general questionnaire asking about sociodemographic, health and lifestyle information (week 15) and the MoBa food frequency questionnaire (FFQ) (weeks 17-22). In addition, participants had to have answered at least one of the 6 questions about organic food (n = 690 excluded), and they had to have reported a credible daily energy intake (>4.5 MJ or <20 MJ) (n = 1065 excluded). This resulted in a sample of 63 808 women (97.3%).

### The MoBa food frequency questionnaire

The MoBa FFQ (http://www.fhi.no/dokumenter/011fbd699d.pdf) is a semi-quantitative food frequency questionnaire designed to capture dietary habits during the first four to five months of pregnancy and has been described in detail elsewhere [24]. Data were collected from February 2002 and onwards. The questionnaire has 40 groups of questions covering the daily intake of 255 specific food items as well as use of organic food in six main food groups (milk/dairy, bread/cereal, eggs, vegetables, fruit and meat).

### Food groups

Based on reported daily intake of 255 specific food items, we selected 58 as well as 28 more aggregated food groups (non-overlapping) for identification of dietary patterns. The interrelations between food groups in the PCA plots were more or less similar independent of which set of food groups that was used as input variables, and the PCA plot with 58 food groups, providing more detail, is only shown in supplemental material. Additional file 1: Table S1 shows the relation between the two sets of food groups, and Additional file 2: Table S2 gives further details of the food items included in the food groups.

Food groups were selected based on significance regarding the quantity and quality of food as well as ability to distinguish between different types of diets. Some food items were not included for their nutritional relevance, but rather as a marker of certain types of foods/diets, for example ketchup as a marker for fast food, soy products as a marker for alternative diets and rice porridge as a marker of traditional foods. Not all of the 225 food items were included in the food groups used in this study, only those judged as relevant according to the above mentioned criteria (186 food groups).

### Consumption of organic food

As in the previous study of organic food consumption in MoBa, the use of organic food was calculated as a sum index based on the question about the frequency of use of organic food specified in six food groups: milk and dairy products, bread and cereal products, egg, vegetables, fruit and meat [22]. The response alternatives for use of organic food were:’never or seldom’,’sometimes’,’often’, or’mostly’ and were given values from 0-3. For those who had answered at least one of the questions about organic food, missing values for one or more of the other questions were interpreted as ‘seldom or never’. The sum index reflects organic food use on a scale ranging from 0 to 18, with 0 representing no use of organic food and 18 representing ‘mostly’ organic for all six food groups. For respondents who had no reported intake of meat (n = 450), eggs (n = 1976), milk/dairy (n = 979) or vegetables (n = 11) and who had not reported organic consumption of the corresponding food group, we upscaled the sum index by multiplying with 6/5 for each omitted food category (additively). This resulted in the upscaling of 1066 subjects, and the re-allocation of 63 respondents into a ‘frequent organic’ consumption group. We defined frequent organic consumption as having a sum index of >6, which corresponds to having reported eating organic food ‘often’ for at least one of the six food categories, given that the answer to all other categories was "sometimes". Consumption of organic food was operationalised in the analyses as ‘no or low’ vs. ‘frequent’ total consumption of organic food (sum index ≤6 vs. >6) and as ‘low’ vs. ‘high’ consumption of the individual six food groups: milk and dairy products, bread and cereal products, egg, vegetables, fruit and meat. The reported frequencies of the six main organic food groups as well as correlations between them have been reported in detail previously [22].

### Statistical analyses

Principal Component Analyses (PCA) were performed to describe the variation in dietary patterns among participants, followed by ANOVA analyses to explore associations between consumption of organic food and dietary patterns (described by the two first principal components) as well as selected foods, beverages and nutrients. The method of first using PCA on the original food consumption data with subsequent ANOVA of the scores to test the effects of the design factors, here: use of organic food, on the scores (PC-ANOVA) is described by Luciano and Næs [25]. We used cross-validation to assess the importance of each component [26]. Since there are many tests in the paper, one should be careful about interpreting each of the values as exact values of significance. This corresponds to the explorative character of the paper with many possibilities tested simultaneously. If wanted, one can look at the Bonferroni p-values, and in for instance Table 1 with 14 different tests for each column, the Bonferroni p-value is equal to 0.0035. As can be seen, there are several values even below that threshold. 

**Table 1:** Personal, socioeconomic and lifestyle characteristics of the study participants and associations with frequent organic consumption and mean dietary patterns scores (n = 63808)

		**All total n**	**Frequent organic consumption**		**Mean score PC1**^**§**^	**Mean score PC2**^**§§**^
			**n**	**%**		
Frequent organic consumption						
	No		58022	90.9	-0.07	0.01
	Yes		5785	9.1	0.66	0.14
Age						
	<20	860	242	28.1	-1.28	-0.24
	20-24	7758	998	12.9	-0.73	-0.08
	25-29	22760	1829	8.0	-0.11	0.01
	30-34	23015	1875	8.1	0.21	0.05
	35-39	8358	723	8.7	0.42	-0.04
	40 +	1053	118	11.2	0.77	-0.21
	Missing data	4				
Prepregnant BMI						
	<18.5	1834	226	12.3	0.01	0.02
	18.5-24.9	40674	3819	9.4	0.16	0.01
	25-29.9	13606	1107	8.1	-0.24	-0.002
	30-34.9	4368	315	7.2	-0.48	-0.03
	35+	1651	129	7.8	-0.55	0.03
	Missing data	1675	190	11.3	0.04	-0.04
Dietary habits						
	Meat/fish	63696	5748	9.0	0.00	0.00
	Vegetarian	112	38	33.9	2.15	0.93
Alcohol in pregnancy						
	No	56391	5101	9.0	-0.04	-0.04
	Yes	7417	685	9.2	0.32	0.28
Smoking in pregnancy						
	No smoking	58462	5144	8.8	0.09	0.01
	Occasionally	1815	219	12.1	-0.54	-0.01
	Daily	3531	426	12.0	-1.21	-0.13
Education						
	<10y-12 y	20460	2260	11.0	-0.64	-0.13
	13-16y	26559	1931	6.9	0.01	-0.03
	17+	15401	1548	10.1	0.84	0.22
	Missing data	1388	147	10.6	0.01	0.12
Student						
	No	57499	4956	8.6	-0.01	0.00
	Yes	6309	830	13.2	0.08	0.04
Household income						
	Low (both<NOK300 000)	18850	2073	11.0	-0.40	-0.13
	Medium (one ≥NOK 300 000)	26456	2137	8.1	-0.05	-0.06
	High (both ≥NOK 300 000)	16625	1277	7.7	0.56	0.26
	Missing data	1887	299	15.9	-0.25	-0.17
Living area						
	Rural	31070	2675	8.6	-0.37	-0.22
	Urban	32492	3079	9.5	0.35	0.21
	Missing data	246	32	13.0	0.52	0.27

^**§**^ High scores on PC1 indicate high intake of vegetables, fruit and berries, wholegrain cereal products and cooking oil, low scores on PC1 indicate high intake of meat, white bread, cakes and sweets.

^**§§**^ High scores on PC2 indicate high intake of pasta, rice, poultry, olive/cooking oil and vegetables, low scores on PC2 indicate high intake of potatoes, lean fish, margarine, fruit and berries.

In this study we wanted to explore the relative amount of the daily intake of the different food groups reported, and they were therefore transformed into shares (g/day of each food group was divided by total g/day eaten for each respondent). This was done in order to eliminate the effect of some participants eating a lot while others eating less. In other words we concentrate on the pattern of the food categories relative to each other. In this process we decided to eliminate the beverages. The main reason for doing so was that the beverages were very dominating in weight as compared to the other food categories. A person with a high intake of beverages and a person who drink very little, but with a similar intake of the other food categories, would then come out as completely different in the data set. This would be an unwanted effect in our study. In addition we wanted to emphasise the relative importance of each food group rather than emphasising those foods which were eaten in larger amount. We therefore used a standardised analysis (we divided each variable by its standard deviation). It is then important to emphasize that we later on when analysing the different food groups, also incorporated the beverages. In this way the beverages are taken care of, but not in such a way that they damage the overall interpretation of the general food intake pattern.

PCA is a method suitable for explorative aims as it is based on few assumptions. The analysis finds the directions with most variability, and projects the information down onto these dimensions. Results from the PCA are presented in two types of interrelated plots: scores plot and loadings plot. From the loadings plot, relations between variables are interpreted: variables (in our case food groups) which are positioned close to each other are highly correlated, while variables on opposite side of the plot are negatively correlated to each other. From the scores plot we can interpret relations between respondents, e.g. those who eat similar diets and those who eat very differently, as well as which food groups that dominate the diet of a particular respondent. A respondent’s score denotes the position in the scores plot, and the position in the scores plot is directly related to the loadings plot: The diet of a respondent who is positioned to the left in the plot is characterised by consumption of food groups to the left in the loadings plot and so on.

Finally, we also performed two sensitivity analyses. First we evaluated the influence of upscaling the sum index of participants who reported no intake of meat, eggs and/or milk/dairy (n = 1066), and second we evaluated the influence of vegetarians (n = 112). Repeating the analysis without these groups resulted in only marginal differences.

PCA analyses were performed using the Unscrambler X version 10.1 (CAMO Software AS, Oslo, Norway). All other analyses were performed using the statistical software PASW statistics 17 (SPSS Inc., IBM Company, Chicago, Ill., USA).

## Results

**Figure 1:**
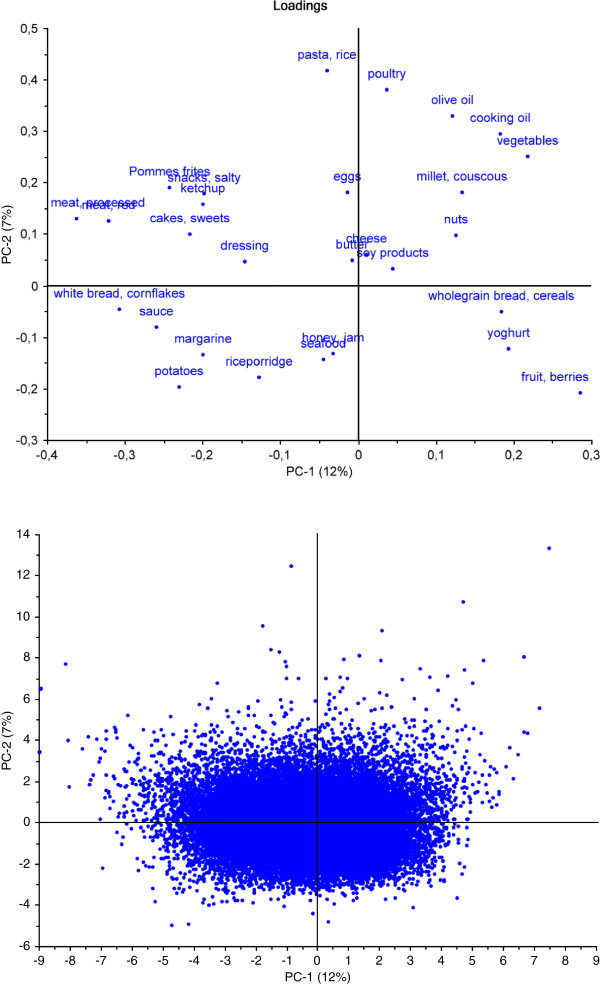
**Loadings and scores plot from PCA (N = 63808).** Names of overlapping food groups in the loadings plot, upper left: processed meat, red meat. Standard deviations for the scores are 1.82 for PC1 and 1.35 for PC2. The explained variance for component 1, 2, 3 and 4 are 12%, 7%, 5% and 5% respectively.

Principal component analyses uncovered relatively large variation in reported dietary behavior among the 63808 pregnant women in this study. The first principal component (PC1), accounting for 12% of the variation in food intake among the women (Figure 1), was characterized by high positive loadings for vegetables, fruit and berries, cooking oil, olive oil, whole grain products and negative loadings for meat, including processed meat, white bread, salty snacks, Pommes frites and cakes and sweets. This principal component was denoted a 'health and sustainability component’. The second principal component (PC2), accounting for 7% of the variation in food intake (Figure 1), was characterized by high positive loadings for pasta and rice, poultry, olive oil, cooking oil, and vegetables and negative loadings for yoghurt, potatoes and fruit. This component did not add significantly to the illumination of dietary variation relevant for health, but rather displayed variation in culinary preferences. A cross-validation based on three segments [26] indicates that more than two components in principle could be assessed as significant. There is, however, a clear indication from the scree plot (i.e explained variance plot, in particular for cross-validation) that the first component is clearly dominating with only relatively minor contributions given by the rest of the components. The third and subsequent principal components were not considered because they were not readily interpretable and added less to explained variation in food intake. 

Frequent consumption of organically produced food, which was reported by 9.1% of the participants, was associated with higher scores on both principal components. While frequent organic consumption did not unambiguously increase with increasing age, education, income and abstinence from alcohol and smoke, the scores for the ‘health and sustainability component’ increased with all of these characteristics (Table 2). When adjusting for all potential confounding variables shown in Table 2, the differences in score between the frequent organic consumers and non-frequent consumers increased from 0.73 to 0.80 for PC1 and from 0.16 to 0.17 for PC2. While the difference between the groups in score on the ‘health and sustainability component’ (PC1) was considerable (0.73 units in the scores plot), and represented approximately 1/10 of the total variation in scores, the difference between the groups in scores on the second principal component was smaller (0.16 units in the scores plot) and in practice insignificant (Table 3). We therefore primarily focus on variation according to the first principal component in the following. Organic consumption of all the six food groups was statistically associated with both principal components, except organic fruit and meat, which were only significantly associated with PC1 (Table 3).

**Table 2:** Differences in food patterns and intake of selected food groups by reported frequency of organic consumption among 63 808 women in the Norwegian Mother and Child Cohort Study 2002-2007

	**Total organic consumption (all food groups combined)**		**Organic consumption by food groups**					
	**Low = sum index≤6**	**Frequent = sum index>6**	**Organic vegetables**	**Organic fruit**	**Organic bread & cereal**	**Organic milk & dairy**	**Organic eggs**	**Organic meat**
**Dietary pattern**	Mean score (SE)	*Difference in patterns scores*						
		Frequent vs. low (SE)	High vs. low organic consumption by food groups (SE)					
PC1(‘health-component’)	-0.07 (0.007)	0.73 (0.03)***	0.82 (0.03)***	0.63 (0.03)***	0.56 (0.03)***	0.59 (0.03)***	0.97 (0.03)***	-0.12 (0.04)**
PC2	0.01 (0.006)	0.16 (0.02)***	0.11 (0.02)***	ns	0.15 (0.02)***	0.13 (0.02)***	0.23 (0.02)***	ns
**Food groups**	Mean intake g/10MJ (SE)	*Difference in intake (g/10MJ)*						
		Frequent vs. low (SE)	High vs. low organic consumption by food groups (SE)					
Vegetables (total)	156 (0.4)	29 (1.4)***	38 (1.5)***	25 (1.6)***	21 (1.6)***	18 (1.5)***	35 (1.4)***	ns
Vegetables (raw)	91 (0.3)	9.0 (1.0)***	16 (1.0)***	8.5 (1.1)***	4.5 (1.1)***	3.6 (1.1)**	15 (1.0)***	-6.2 (1.4)***
Fruit & berries	279 (0.8)	44 (2.6)***	55 (2.9)***	61 (3.1)***	31 (3.1)***	21 (2.9)***	43 (2.6)***	10 (3.8)**
Nuts	2.3 (0.02)	1.1 (0.1)***	0.9 (0.1)***	0.6 (0.1)***	1.1 (0.1)***	0.7 (0.1)***	1.5 (0.1)***	-0.2 (0.1)*
Pasta & rice	51 (0.1)	4.5 (0.5)***	5.4 (0.5)***	5.3 (0.6)***	3.9 (0.6)***	2.9 (0.5)***	2.7 (0.5)***	4.0 (0.7)***
White bread & cornflakes	118 (0.4)	-15 (1.3)***	-16 (1.5)***	-9.8 (1.6)***	-14 (1.6)***	-12 (1.5)***	-23 (1.3)***	ns
Wholegrain bread/cereal	131 (0.4)	9.4 (1.5)***	9.5 (1.6)***	ns	101 (1.8)***	8.2 (1.7)***	21 (1.5)***	15 (2.1)***
Cakes and sweets	104 (0.2)	-7.7 (0.7)***	-7.5 (0.7)***	-8.2 (0.8)***	-8.5 (0.8)***	-7.0 (0.7)***	-6.1 (0.7)***	-6.6 (0.9)***
Meat (total)	169 (0.2)	-24 (0.8)***	-22 (0.9)***	-20 (0.9)***	-21 (0.9)***	-23 (0.9)***	-22 (0.8)***	-8.1 (1.1)***
Red meat	102 (0.2)	-15 (0.6)***	-14 (0.7)***	-13 (0.7)***	-14 (0.7)***	-15 (0. 7)***	-15 (0.6)***	-1.8 (0.9)*
Processed meat	41 (0.1)	-7.2 (0.3)***	-7.7 (0.3)***	-6.4 (0.3)***	-5.9 (0.3)***	-6.1 (0.3)***	-7.5 (0.3)***	-2.7 (0.4)***
Eggs	112 (0.1)	1.7 (0.2)***	1.5 (0.2)***	1.2 (0.2)***	1.2 (0.2)***	1.1 (0.2)***	4.1 (0.2)***	1.0 (0.3)***
Yoghurt	80 (0.5)	14 (1.6)***	16 (1.7)***	15 (1.8)***	11 (1.8)***	12 (1.7)***	18 (1.6)***	ns
Lowfat milk	312 (1.2)	-37 (4.4)***	-39 (4.6)***	-28 (4.8)***	-42 (4.9)***	ns	-42 (4.1)***	-17 (5.9)**
Softdrinks	156 (1.0)	ns	-11 (3.6)**	ns	ns	ns	-28 (3.3)***	44 (4.7)***
Softdrinks diet	156 (1.3)	-43 (4.6)***	-36 (5.1)***	-35 (5.3)***	-35 (5.4)***	-54 (5.1)***	-47 (4.6)***	ns
Tea	183 (1.1)	35 (3.7)***	39 (4.1)***	27 (4.3)***	34 (4.4)***	24 (4.1)***	52 (3.7)***	ns

* p-value < 0.05, ** p-value < 0.01, *** p-value < 0.001. One-way ANOVA analyses.

**Table 3:** Differences in intake of selected nutrients by reported frequency of organic consumption among 63 808 women in the Norwegian Mother and Child Cohort Study 2002-2007

	**Total organic consumption (all food groups combined)**		**Organic consumption by food groups**					
	**Low = sum index≤6**	**Frequent = sum index>6**	**Organic vegetables**	**Organic fruit**	**Organic bread & cereal**	**Organic milk & dairy**	**Organic eggs**	**Organic meat**
**Nutrients**	Mean intake (SE)	*Difference in intake*						
		Frequent vs. low (SE)	High vs. low organic consumption by food groups (SE)					
Energy kJ/day	9686 (11)	592 (36)***	577 (39)***	681 (42)***	556 (42)***	560 (40)***	361 (36)***	646 (51)***
E% from protein	15.5 (0.009)	-0.3 (0.03)***	-0.2 (0.03)***	-0.3 (0.03)***	-0.3 (0.03)***	-0.2 (0.03)***	-0.1 (0.03)***	-0.4 (0.04)***
E% from fat	30.4 (0.02)	0.2 (0.1)**	ns	-0.3 (0.1)***	0.3 (0.1)**	0.4 (0.1)***	0.3 (0.1)***	ns
E% from carbohydrate	53.9 (0.02)	ns	0.2 (0.1)**	0.5 (0.1)***	ns	-0.2 (0.1)**	-0.2 (0.1)**	ns
E% from added sugar	10.6 (0.02)	ns	-0.3 (0.1)**	ns	ns	ns	-0.5 (0.1)***	0.8 (0.1)***
Dietary fiber g/10MJ	31.8 (0.03)	1.5 (0.1)***	2.0 (0.1)***	1.4 (0.1)***	1.3 (0.1)***	0.5 (0.1)***	2.0 (0.1)***	0.7 (0.2)***
β-carotene μg/10MJ	2702 (7.7)	293 (26)***	520 (28)***	294 (30)***	230 (30)***	150 (29)***	316 (26)***	ns
Vitamin D, μg/10MJ	3.56 (0.009)	0.2 (0.03)***	0.2 (0.03)***	0.1 (0.03)**	0.1 (0.04)**	ns	0.2 (0.03)***	ns
Vitamin C mg/10MJ	171 (0.3)	16 (1.2)***	21 (1.3)***	21 (1.3)***	9.4 (1.4)***	7.2 (1.3)***	17 (1.2)***	6.1 (1.7)***
Folate, μg/10MJ	284 (0.3)	21 (1.0)***	26 (1.1)***	23 (1.1)***	15 (1.2)***	13 (1.1)***	25 (1.0)***	4.5 (1.4)**
Calcium, mg/10MJ	1075 (1.3)	ns	ns	ns	-13. (5.0)**	30 (4.7)***	ns	-20 (6.1)**
Magnesium, mg/10MJ	414 (0.2)	7.4 (0.8)***	11 (0.9)***	6.6 (0.9)***	4.8 (1.0)***	3.9 (0.9)***	14 (0.8)***	9.2 (1.2)***
Potassium, mg/10MJ	4169 (2.9)	90.6 (9.7)***	162 (11)***	135 (11)***	42 (12)***	36 (11)**	128 (9.7)***	41 (14)**
Sodium, mg/10MJ	3190 (2.3)	-127 (6.8)***	-118 (7.5)***	-140 (7.9)***	-119 (8.0)***	-118 (7.6)***	-100 (6.8)***	-97 (9.7)***

* p-value < 0.05, ** p-value < 0.01, *** p-value < 0.001. One-way ANOVA analyses.

These findings were corroborated in the analyses which showed increases in daily intake of food groups characterizing the components, e.g. vegetables (+29 g/d), fruit and berries (+44 g/d), whole grain products (+9.4 g/d), pasta and rice (+4.5 g/d), and decreases in intake of meat (-24 g/d), processed meat (-7.2 g/d), white bread (-15 g/d) and cakes and sweets (-7.7 g/d) among women with frequent consumption of organic food (Table 3). In addition to a higher total vegetable intake, women with frequent organic food consumption ate a larger proportion of raw vegetables (+9 g/day). Regarding beverages, frequent organic consumption was associated with less artificially sweetened soft drinks (-43 g/10 MJ), (but not sugar sweetened soft drinks), less low fat and skimmed milk (-37 g/10 MJ), and more tea (+35 g/10 MJ).

We observed some variations in dietary patterns associated with consumption of the six different groups of organically produced foods (Table 3). The increase in vegetable consumption was particularly high for high consumption of organic vegetables, and the highest increase in consumption of fruit and berries was found among participants with high consumption of organic fruit and berries. High consumption of organic meat represented an exception from the overall associations between frequent organic consumption and dietary patterns: no increase in vegetable consumption, only a small increase in fruit and berries, no decrease in intake of white bread and highly processed breakfast cereals, and only a small reduction in meat intake. Consumption of organic meat was also associated with consumption of more sugar-sweetened soft drinks, and no difference in intake of artificially sweetened soft drinks, contrary to organic consumption of the other five food groups.

**Figure 2:**
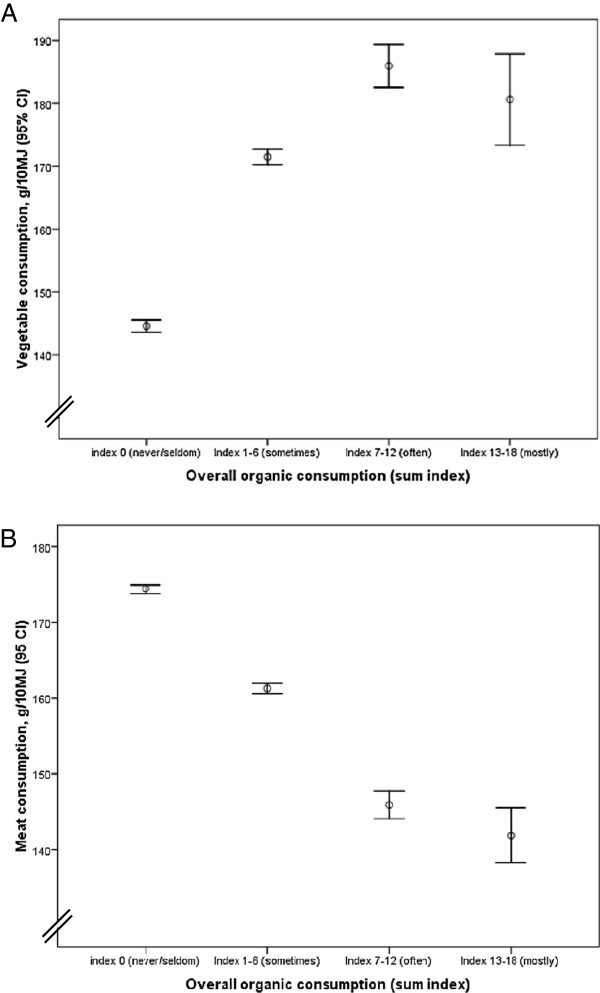
**A and B.** Amount of vegetables and meat in the diet according to consumption of organic food.

A gradual increase in vegetable consumption and a corresponding decrease in meat consumption were evident across increasing frequencies of reported organic consumption (Figure 2). The graphs depict a significant difference in consumption of both meat and vegetables already at the lowest frequency of organic consumption, and that these differences become more pronounced with increasing consumption of organically produced food. Even among women with high scores on PC1 (e.g. high vegetable consumption and low meat consumption), frequent consumption of organic food was associated with higher vegetable consumption and reduced meat consumption compared with women within the same pattern score but with low organic consumption (data not shown).

The results show that rather than a mere ‘substitution’ of conventionally produced foods with organically produced foods of similar types (substituting conventional minced meat with organic minced meat, for example), we see qualitative differences in dietary composition. These qualitative differences include the relative amounts of main food groups (such as less meat and more vegetables), as well as emphasis on different types of products (if meat: rather lamb than sausages,) and food preparation and degree of processing (more vegetables eaten raw*,* more whole foods rather than highly processed) (see Table 3, Figure 1 and Additional file 3: Figure S1).

Frequent organic consumption and high consumption of all organic food groups were associated with higher energy intake (Table 1). The same was found for the relative contribution of energy from protein. However, the differences in energy contributed by the other macronutrients were small. Furthermore, higher intakes of vegetables, wholegrain products, fruit and berries and lower intakes of processed foods among the frequent organic consumers were reflected in higher relative (energy adjusted) intakes of beta-carotene, ascorbic acid, folate, fiber and magnesium and lower intakes of sodium (Table 1). There were only marginal differences in intake of zinc, selenium and iron between the groups (results not shown).

## Discussion

The main finding of this study was that frequent consumption of organic food among the pregnant women in the MoBa study was strongly associated with higher scores on the ‘health and sustainability component’ (PC1), i.e. a diet characterized by more vegetables, fruit and berries, and whole grain cereal products and less meat, including processed meat, white bread, refined cereal products, cakes and sweets, compared with participants with no or low consumption of organic food. Diets of the pregnant women with frequent organic consumption, consequently, were more in line with dietary recommendations for health as well as ecological sustainability.

In the multivariate part of the present study we have considered data based on first adjusting for amount of food eaten in order to highlight pattern rather than amount. We also decided to exclude beverages when extracting underlying dietary components (patterns) since they had strong dominance on the results. Thirdly we used standardization in order to give all the foods equal opportunity to contribute meaning which means that we consider relative differences in each of the foods rather than the dominating effect of those with the largest variance. This analysis revealed clear differences between ‘healthy’ and ‘unhealthy’ diets represented in particular by the score along the first principal component. In other studies of dietary patterns, contrasting patterns are often found, the first two typically being ‘prudent’ and ‘western’ (or denoted with similar names) [18,27-29]. In the present study, however, both ‘healthy’ and ‘unhealthy’ diets are covered by the first principal component, represented by high and low scores, respectively. This dual dimension within the first component was also seen in a large cohort of pregnant women in Southampton [30,31]. The first principal component extracted in that study was characterized by high intakes of healthy food items vs. unhealthy food items.

In the present study, two types of food issues were brought together: *How* food is produced and *what kinds of* foods that are eaten. The findings confirm that in the dietary practices of the pregnant women in this study, there was an association between these two food dimensions. We have no means of knowing the order in which choices were made and practices established: Did the choice of buying organically produced food form the choice of diet, or did more people who preferred certain types of diets choose organically produced foods – or did reflection about both these aspects of food mutually influence each other? Regardless of how these practices have developed, the significant differences in dietary characteristics are of relevance with regard to their ability to contribute to good health and ecological sustainability. Dietary recommendations aiming at both these goals are to a large extent overlapping. Central to ecological sustainability is eating lower in the biological food chain (i.e. more plant foods and less meat), because it maximizes the amount of high quality food for human nutrition relative to the ecological costs of production, and this also fits well with health advice.

We have previously reported a large difference in prevalence of vegetarians (+23 percentage points) among the pregnant women in MoBa who frequently ate organic food compared to those who did not [22]. Since the total number of vegetarians was very low, however, (0.2%, ibid) the practical significance of the change in dietary pattern expressed as a shift of the whole group of frequent organic consumers from lower to higher scores on the ‘health and sustainability component’ (PC1) is by far much larger. An implication of this was that on average, the frequent organic consumers in the MoBa-study had a reported intake of over half a kilo more vegetables, fruit and berries weekly than those with low organic consumption, and they reached the Norwegian recommendations of ‘5 a day’ (500 g vegetables, fruit and berries a day), while those with no or little organic food in their diets were 65 g short of doing so.

In addition to a higher total consumption of vegetables, women with frequent organic consumption also ate a larger proportion of these vegetables raw. Higher intakes of ‘whole foods’ such as raw vegetables, fruit and berries, nuts, wholegrain bread and cereal products among the frequent consumers of organic food in the present study are in line with preferences for less processed and refined foods as reported by both Holt [32] and Torjusen [33], as well as consumers’ associations of organic food with characteristics such as ‘natural’ and ‘home made’ [16,34]. Similarly, lower consumption of refined and processed foods such as processed meat, highly refined cereal products, potato chips (‘salty snacks’) and Pommes frites further confirms such trends. Characteristic in this respect is also the negative correlation between tomato ketchup and fresh tomatoes, as representatives of diets with ‘fast food’ or processed food vs. diets based on fresh produce and whole foods (see details in Additional file 3: Figure S1).

There was large variation in use of different types of fat in the diet (Figure 1), and the association between frequent consumption of organic food and more use of olive oil and cooking oil, which is in line with previous findings [33], may be related to a larger emphasize on health among those who choose organic food.

The observed negative correlation between processed meat and meat from intensive production systems (such as pork) with negative loadings and meat from extensive production (lamb), with positive loadings on the ‘health and sustainability component’ (Additional file 3: Figure S1), may be interpreted in light of ecological consciousness or a preference for what is perceived as more ‘natural’ or ‘closer to nature’.

In her study of dietary habits among organic consumers in the UK, Holt reported a shift away from a diet focused on meat, potato and bread, towards increased consumption of vegetable foods and in particular, the incorporation of non-traditional plant foods, such as nuts, pulses and grains into the diet, resulting in a greater diversity of protein and staple foods among consumers of organic foods [13]. In the present study, frequent consumers of organic food had slightly lower percentage of energy derived from protein (Table 1), as well as a higher relative proportion of proteins from plant foods rather than animal foods, but still a protein intake well within (and in the upper levels of) dietary recommendations for pregnant women [35]. Higher intakes of foods such as nuts, pulses and legumes and soy products represent alternative sources of proteins in diets with less meat. We also recognize more diversity in ‘staple foods’: higher intake of less traditional grains such as millet, as well as higher intake of pasta and rice (Figure 1 and Table 3).

Our finding of higher average energy intake among frequent organic consumers (about 600 kJ/day more, Table 1) is likely to be related to higher levels of physical activity, as we have previously found that a larger proportion of the frequent organic consumers in the MoBa-study exercised regularly and were normal or low weight compared to those with no or low organic consumption [22]. Their food intake therefore seems to be more in balance with their energy expenditure.

The large sample size including women from both urban and rural regions, representing all age groups and socio-economic groups is a major strength in the present study. The large sample size ensures large variation in dietary composition and use of organically produced food. However, only 38.5% of those who were invited participate in MoBa, which imply that the prevalence of organic consumption may not be representative for all pregnant women in Norway. The potential influence of self-selection in MoBa has been evaluated, and no statistically relative differences in association measures were found between participants and the total population regarding eight exposure-outcome associations [36]. Hence, the selection bias in MoBa is not likely to influence the associations between reported use of organic food and dietary behavior among the frequent organic consumers. The sum index is a relatively crude measure, but in spite of this we observed important associations between overall frequent organic consumption and dietary quality. We also observed differences in food and nutrient intakes between frequent consumers of the various organic food groups. When designing a new FFQ, as was done for the MoBa cohort, there is a trade-off between the number of questions and the burden imposed on respondents. If questionnaires ask in too much detail there is an increased risk of participant drop-out [37,38]. We do not know how the MoBa FFQ influenced the participation rate, but 93% of the women participating in MoBa did answer the FFQ [21].

Misreporting is a serious error in all dietary assessment methods [39], and it has been shown that foods perceived as ‘unhealthy’ are underreported to a larger degree than foods perceived as ‘healthy’ [40]. The food frequency methods challenges participants with complex cognitive tasks and is particularly difficult to answer early in pregnancy when many women are experiencing nausea and changes in appetite and eating patterns. However, the MoBa FFQ was developed and validated for use in pregnancy [24] and the validation study demonstrated that relative to a dietary reference method and several biological markers, it produces a realistic estimate of the habitual intake and is a valid tool for ranking pregnant women according to high and low intakes of energy, nutrients, and food [41].

We have previously reported that frequent consumption of organic food among pregnant women in the MoBa-study was not solely associated with socio-economic and lifestyle factors that are normally associated with good health [22]. While women who consumed organic food exercised more frequently, a larger proportion of them also consumed alcohol and smoked during pregnancy (although total prevalences were low). Lower household income and lower as well as higher levels of education were associated with frequent organic food consumption. While the consumption of organic food was not typically associated with high socio-economic status and healthy lifestyle factors, the association between frequent organic consumption and diet quality appeared to be clearer: pregnant women who chose organically produced food also ate diets that were more in line with health recommendations. Dietary quality is especially critical in pregnancy and PCA-derived dietary patterns in pregnancy have been associated with various health outcomes in well-nourished populations, including gestational weight gain [42]), preeclampsia [18], foetal growth [43] and post-partum depression [44]. There are, however, few previous studies addressing the combination of organic food consumption and the general dietary patterns and quality among pregnant women.

A methodological implication of the present study is that information about the diet in general needs to be included in future studies of possible health outcomes related to organic food consumption. It will further be advantageous to have specific information about consumption of organic food of different food groups, since high consumption of organic meat was associated with a somewhat different dietary pattern than that of organic vegetables, fruit, cereal products, dairy products and eggs.

Large knowledge gaps remain in our understanding of the consumption of organic food, related food practices and possible implications for health of mother and child. It remains an unsettled question whether there are systematic differences in the absolute content of nutrients and other substances of importance for health in foods that are organically or conventionally produced. The food analyses in the present study are based on the same food tables for both organically and conventionally produced foods, because at present there are no separate data on organically produced foods in the form of systematic food tables. If such differences are present, estimates of nutrient intakes would be imprecise or wrong. There are knowledge gaps in our understanding of how the consumption of organic food may be related to health, both in short and long term. Findings that indicate possible associations between consumption of organic food and health, such as prevalence of allergic diseases among children in families with organic consumption [45,46], will be addressed in future studies.

## Conclusions

The present study showed that pregnant Norwegian women reporting frequent consumption of organically produced food had a dietary pattern and quality more in line with public advice for healthy and sustainable diets. A methodological implication is that the overall diet needs to be included in future studies of potential health outcomes related to consumption of organic food during pregnancy.

## Competing interests

The authors declare that they have no competing interests.

## Authors’ contributions

HMM took the initiative and was PI of the study. HT and HMM designed the study. HT performed the statistical analyses and wrote the manuscript. TN, GL, ALB and MH assisted in the statistical analyses. All authors contributed to the interpretation of results and read and approved the final manuscript.

## Pre-publication history

The pre-publication history for this paper can be accessed here:

http://www.biomedcentral.com/1471-2458/12/612/prepub

## Supplementary Material

Additional file 1 **Table S1.** Overview of food groups in PCA (Figure 1 and 2). Click here for file

Additional file 2 **Table S2.** Overview of food items from the FFQ included in food groups. Click here for file

Additional file 3 **Figure S1.** Loadings plot from PCA with 58 food groups (N=63808). Click here for file
