# Supplementary material for: Food patterns and dietary quality associated with organic food consumption during pregnancy; data from a large cohort of pregnant women in Norway
Source: BMC Public Health. 2012 Aug 6;12:612. doi: 10.1186/1471-2458-12-612 (PMC3490940; doi:10.1186/1471-2458-12-612)
Supplement: Additional file 1 — Table S1. Overview of food groups in PCA (Figure 1 and 2). [file 1471-2458-12-612-S1.doc]

## SUPPLEMENTAL Table 1. Overview of food groups in PCA (Figure 1 and 2)

|  | 58 Food groups (Figure 1) | 28 Food groups (Figure 2) |
| --- | --- | --- |
| 1 | Cruciferous vegetables, onions, tomatoes, mushrooms, corn, green leafy vegetables, root vegetables, legumes and pulses, other vegetables | Vegetables |
| 2 | Potatoes (boiled, baked, mashed), creamed potatoes, potato casserole | Potatoes |
| 3 | Pommes frites | Pommes Frites |
| 4 | Citrus, Nordic fruit, temperate fruit, tropical fruit, banana, dried fruit, berries | Fruit and berries |
| 5 | Nuts | Nuts |
| 6 | Dark bread, crisp bread, wholegrain cereals | Wholegrain bread and cereals |
| 7 | White bread, cornflakes | White bread and cornflakes |
| 8 | Pasta and rice | Pasta, rice and millet |
| 9 | Millet and couscous | Millet and couscous |
| 10 | Rice porridge | Rice porridge |
| 11 | Waffles and buns, cakes and biscuits, dairy desserts, chocolate and sweets | Cakes and sweets |
| 12 | Salty snacks | Salty snacks |
| 13 | Cheese | Cheese |
| 14 | yoghurt | Yoghurt |
| 15 | Eggs | Eggs |
| 16 | Lean fish, oily fish, shellfish, fish spread | Seafood |
| 17 | Poultry | Poultry |
| 18 | Pork, beef, lamb, venison, offal, meat spread | Red meat and pork |
| 19 | Processed meat, Pizza and taco | Processed meat |
| 20 | Honey and jam | Honey and jam |
| 21 | Olive oil | Olive oil |
| 22 | Cooking oil | Cooking oil |
| 23 | Butter | Butter |
| 24 | Margarine | Margarine |
| 25 | Ketchup | Ketchup |
| 26 | Dressing | Dressing |
| 27 | Sauce | Sauce |
| 28 | Soy products | Soy products |
