# Supplementary material for: Food patterns and dietary quality associated with organic food consumption during pregnancy; data from a large cohort of pregnant women in Norway
Source: BMC Public Health. 2012 Aug 6;12:612. doi: 10.1186/1471-2458-12-612 (PMC3490940; doi:10.1186/1471-2458-12-612)
Supplement: Additional file 2 — Table S2. Overview of food items from the FFQ included in food groups. [file 1471-2458-12-612-S2.doc]

## SUPPLEMENTAL Table 2. Overview of food items from the FFQ included in food groups

|  | Foods or food groups | Food items in the FFQ |
| --- | --- | --- |
| 1 | Cruciferous vegetables | Cauliflower, broccoli, cabbage, brussels sprouts |
| 2 | Onions | Garlic, onion, leek, spring onion |
| 3 | Tomatoes | Tomatoes |
| 4 | Mushrooms | Mushrooms (champignon, wild mushrooms) |
| 5 | Corn | Corn |
| 6 | Green leafy vegetables | Green salad (lettuce, ruccula, endives, spinach, chicory), |
| 7 | Root vegetables | Carrot, swede |
| 8 | Legumes and pulses | Green beans, green peas, dish with lentils/beans |
| 9 | Other vegetables | Frozen vegetables, aubergine, zucchini, bell peppers, celery, avocado |
| 10 | Potatoes | Potatoes (boiled, baked, mashed), Creamed potatoes, potato casserole |
| 11 | Pommes frites | French fries, fried potatoes |
| 12 | Citrus | Orange, mandarin, grapefruit |
| 13 | Fruit, nordic | Apple, pear, plum |
| 14 | Fruit, temperate | Grapes, peach/nectarine, melon, other (kiwi) |
| 15 | Fruit, tropical | Papaya, mango |
| 16 | Banana | Banana |
| 17 | Fruit, dried | Dried apricot, raisin, prune, fig, date |
| 18 | Berries | Strawberries, other berries (blueberries) |
| 19 | Nuts | Peanuts, other nuts (almonds, hazel nuts, cashew nuts) |
| 20 | Dark bread | Fiber bread, whole-grain bread |
| 21 | Crisp bread | Crisp bread, rye crisp, rusk |
| 22 | Wholegrain cereals | High grain cereals (müsli), oat flakes, oat flake porridge |
| 23 | White bread | White bread, low fiber bread, baguettes, ciabatta |
| 24 | Corn flakes | Corn flakes |
| 25 | Pasta | Spaghetti, macaroni, noodles |
| 26 | Rice | Rice (normal, whole) |
| 27 | Millet and couscous | Millet, couscous |
| 28 | Rice porridge | Rice porridge/rice pudding |
| 29 | Waffles and buns | Waffles and pancakes, sweet buns and rolls |
| 30 | Cakes and biscuits | Danish pastry, doughnut, sponge cake, chocolate cake, cream layer cake, sweet biscuits, |
| 31 | Dairy desserts | Ice-cream, yoghurt ice-cream, chocolate pudding, custard |
| 32 | Chocolate and sweets | Plain chocolate, fancy and filled chocolate, caramel, candies, liquorice, jelly sweets, marshmallow, marzipan, pastille with sugar, pastille sugar free |
| 33 | Salty snacks | Potato chips, popcorn |
| 34 | Cheese | Whey cheese goat milk, hard cheese, cream cheese, blue cheese, other kinds of cheese, regular and low fat |
| 35 | Yoghurt | Yoghurt full-fat and low-fat |
| 36 | Eggs | Eggs, raw, cooked, scrambled |
| 37 | Lean fish | Cod, saithe, haddock, Pollock, plaice, flounder, tuna, perch, pike, cat fish, fish burger, fish soufflé |
| 38 | Fatty Fish | Mackerel, herring, salmon, trout |
| 39 | Shellfish | Shrimps, crab, mussels |
| 40 | Fish spread | Liver and roe spread, mackerel/sardine in tomato sauce, sardine in oil, herring, pickled, shrimp, crab, |
| 41 | Poultry | Chicken and turkey fillet, chicken and/or turkey sausage, pan fried/ baked/boiled chicken or turkey, chicken schnitzel, nuggets, other poultry (duck, goose, ostrich) |
| 42 | Pork | Pork |
| 43 | Beef | Beef, veal |
| 44 | Lamb | Lamb, mutton |
| 45 | Venison | Venison |
| 46 | Offal | Liver, kidney, other offal, hashed lungs |
| 47 | Meat spread | Ham, roast beef, cold cuts of lamb, calf, salami, Swedish sausage, liver pâté, |
| 48 | Processed meat | Meat sauce for pasta dishes, sausages, hot-dogs, meat balls, meat loaf, hamburger, meat patty, minced meat, beef/pork/lamb stew |
| 49 | Pizza and taco | Pizza, frozen and homemade, taco |
| 50 | Honey and jam | Jam, honey |
| 51 | Olive oil | Olive oil |
| 52 | Cooking oil | Soya oil, sunflower oil, rape seed oil, corn oil |
| 53 | Butter | Butter, low-fat butter, melted butter |
| 54 | Margarine | Normal margarine, low-fat margarine |
| 55 | Dressing | Spread with mayonnaise (Italian etc.), Mayonnaise, remoulade, dressing, regular and low-fat (Thousand-island etc.) |
| 56 | Sauce | Sauce (béchamel or gravy, Bearnaise) |
| 57 | Ketchup | Tomato ketchup |
| 58 | Soy products | Soy products (dinner) |
