# Supplementary material for: Food patterns and dietary quality associated with organic food consumption during pregnancy; data from a large cohort of pregnant women in Norway
Source: BMC Public Health. 2012 Aug 6;12:612. doi: 10.1186/1471-2458-12-612 (PMC3490940; doi:10.1186/1471-2458-12-612)
Supplement: Additional file 3 — Figure S1. Loadings plot from PCA with 58 food groups (N=63808). [file 1471-2458-12-612-S3.doc]

## SUPPLEMENTAL Figure 1. Loadings plot from PCA with 58 food groups (N=63808)

Names of overlapping food groups in the loadings plot:

Upper left: beef, pizza, pommes frites, salty snacks

Upper right: onions, leafy vegetables

Central right: oily fish, lamb, soy products

Central: crisp bread, offal

Lower left: citrus, banana

Lower left: margarine, meat spread
